# Mapping the technology–demand interface: how brain organoid evolution is reshaping the translational landscape of stroke

**DOI:** 10.3389/fcell.2026.1983701

**Published:** 2026-10-20

**Authors:** Caohang Qiu, Ning Sun, Jun Yang, Ling Tan, Maoyu Zhao, Yuyao Fu, Gonghong Liu, Zhifu Song, Wenyou Wu

**Affiliations:** 1 Department of Cerebrovascular Diseases, Luzhou People’s Hospital, Luzhou, China; 2 Department of Pediatric Surgery, Sichuan Provincial Women’s and Children’s Hospital, Chengdu, China; 3 Department of Pediatric Surgery, The Affiliated Women’s and Children’s Hospital of Chengdu Medical College, Chengdu, China

**Keywords:** assembloids, brain organoids, electrophysiology, organoid-on-a-chip, oxygen–glucose deprivation, stroke

## Abstract

Stroke research faces a dual modeling barrier: interspecies divergence and oversimplified 2D cultures have contributed to the failure of many neuroprotective trials. Brain organoids, which provide human genetic backgrounds and 3D neural networks, offer a promising but incomplete solution. Existing reviews often address isolated technological aspects without linking them to stroke-specific demands. This narrative review establishes a “technological iteration–clinical demand” mapping matrix (Figure 1). We trace organoid evolution from static oxygen–glucose deprivation models to vascularized, assembloid, and microfluidic chip-integrated systems, and toward multi-organ coupling. We highlight how these advances may uncover human-specific neuroinflammation, proposed PANoptosis-like spatiotemporal cascades, and synaptic deficits mediated by human-specific genes such as SRGAP2C. We argue that the inability of animal models to resolve certain human-specific targets is driving organoids from static endpoint models toward dynamic platforms with perfusable vasculature and immune integration, although current systems remain at an early stage. We critically evaluate the preclinical role of organoids, defining scenarios where they may partially replace animal testing and where they cannot. Major bottlenecks include fetal-like organoid maturity (transcriptome approximately equivalent to gestational weeks 12–20) versus the average stroke onset age of about 70 years, and the absence of peripheral immune infiltration, which mediates a large fraction of secondary injury. We outline potential solutions including multi-omics integration, inducible aging, gene editing, and multi-organ chip coupling, and emphasize that standardized evaluation frameworks are needed to translate organoid findings into personalized stroke therapy.

## 1 Introduction

### 1.1 Systemic limitations of current stroke models

Over the past 2 decades, more than 1,000 neuroprotective strategies validated in animal models have largely failed in clinical trials, with a translational success rate of less than 5%. Systematic analyses of these failures underscore the severity of the modeling crisis (Shi et al., 2018; O’Collins et al., 2006; Dirnagl and Endres, 2014; Fisher et al., 2009). This impasse reflects two fundamental modeling deficiencies. First, the interspecies gap. Rodent and human brains diverge in white matter proportion, cortical folding patterns, neurovascular unit cellular ratios, and glial immune signaling (TREM2, complement, inflammasome circuits) (Ventura-Antunes et al., 2013). Human astrocytes differ from murine astrocytes in morphology, territorial organization, and functional signaling; broad claims of a fixed 10-fold size difference are not uniformly supported and should be replaced by more precise species-specific comparisons. NMDA receptor antagonists showed robust rodent efficacy yet collectively failed in human trials—not merely because of GluN2A/GluN2B ratio differences, but because human excitotoxic signaling depends more heavily on scaffolding complexes such as PSD-95/nNOS, which rodent models inadequately capture (Ma et al., 2026). Non-human primates, despite closer metabolic and structural homology, are impractical for routine screening because of injury non-uniformity, behavioral endpoint heterogeneity, and prohibitive ethical and cost barriers (Nishimuta et al., 2011). Standardized preclinical guidelines have long emphasized the need to test agents in multiple species, ages, and comorbid conditions—standards rarely met in practice (Dirnagl and Endres, 2014; Fisher et al., 2009).

Second, 2D culture limitations. Monolayer neurons lose *in vivo* polarity; glia exist in non-physiological, chronically activated states incapable of forming functional synaptic networks. Stroke fundamentally involves neurovascular unit collapse—neurons, astrocytes, microglia, pericytes, endothelial cells, and extracellular matrix—within 3D space. 2D systems cannot recapitulate oxygen–glucose gradients, immune migration, dynamic vascular–glial interactions, or cortical spreading depolarization, all critical pathological events (Lombardozzi et al., 2025; Santiago et al., 2023).

This exposes the central paradox: systems retaining human genetic background sacrifice tissue architecture; systems preserving 3D organization sacrifice human background. Recent advances in self-assembling brain organoids, vascularized organoids, and organoid–microfluidic integrated systems have enabled more complex reconstruction of human neurovascular features *in vitro*, providing additional tools to investigate limitations of conventional models. However, these systems remain incomplete and should not be presented as a complete replacement for animal models.

### 1.2 The rise of brain organoids

Brain organoids originated from directed 3D self-organization of pluripotent stem cells: the SFEBq method (2008) first induced polarized cortical-like structures (Eiraku et al., 2008); Matrigel embedding with spinning bioreactors (2013) expanded neuroepithelial tissues to millimeter scale, marking the formal birth of cerebral organoids (Lancaster et al., 2013). Subsequent advances include regionally specific induction, multi-lineage co-culture, and dynamic chip perfusion with bioreactor control (Xiang et al., 2021; Sidhaye and Knoblich, 2021). Since 2023, assembloid–microfluidic strategies have enabled primitive perfusable vascular-like networks in cortical organoids. In this review, “assembloid” refers to the controlled fusion of organoids with different regional identities to study inter-regional connectivity; “organoid-on-a-chip” refers to organoids cultured in microfluidic devices that provide controlled perfusion, oxygen gradients, and/or mechanical cues.

Core advantages are threefold: (1) human origin—induced pluripotent stem cell (iPSC)-derived organoids carry patient-specific genetic backgrounds, making individual genetic contributions to stroke susceptibility experimentally tractable; (2) 3D functional networks—mature cortical organoids generate synchronized calcium oscillations, exhibit laminar differentiation, and establish excitation–inhibition balance, underpinning capacity to model post-stroke network dysfunction (Kawano et al., 2025; Wang et al., 2020); (3) observable developmental dynamics. Current organoids remain transcriptionally fetal, and this “immaturity ceiling” is a major limitation for modeling adult stroke.

### 1.3 Distinct contribution: a “technological iteration–demand coupling” mapping matrix

Existing reviews catalogue vascularization engineering, regional specification methods, or microfluidic parameters in isolation, narrating technological progress along independent trajectories rather than embedding it within stroke research’s authentic demand network (Rizzuti et al., 2024; Halkoluoto et al., 2026). We establish a mapping matrix (Figure 1) that directly couples each technological advance to multi-layered stroke research demands, stratified into four hierarchical levels: (1) basic pathology—faithful recapitulation of ischemic–hypoxic and excitotoxic core events; (2) tissue microenvironment—multi-cellular vascular–immune–neural interactions; (3) remote network—secondary damage in distant brain regions; (4) translational utility—perfusion, drug administration, and transplantation. Taking these four demand layers as the vertical axis and organoid engineering strategies/iterations—static 3D → vascularized/regionalized → chip-integrated dynamic → multi-organ coupling—as the horizontal axis, we systematically examine progress and gaps at each intersection. The matrix is a qualitative framework rather than a formal meta-analysis. To improve transparency, each rating was determined by integrating three predefined dimensions: (i) availability of direct experimental evidence supporting the platform’s ability to address the specific stroke-related demand, (ii) reproducibility of the reported capability across independent studies or experimental settings, and (iii) degree of functional matching between the engineering feature and the corresponding biological requirement. Ratings were assigned by author consensus based on these criteria and the available literature. “Low” indicates limited, indirect, or largely conceptual evidence; “Moderate” indicates partial capability with substantial limitations; and “High” indicates that the platform has demonstrated relatively strong capability in addressing the corresponding demand under at least some validated conditions, while not implying complete biological recapitulation. Figure 1 treats platforms as historical engineering iterations, whereas Figures 2 and 3 emphasize that the optimal future platform will likely combine these strategies rather than replace them sequentially.

**FIGURE 1:**
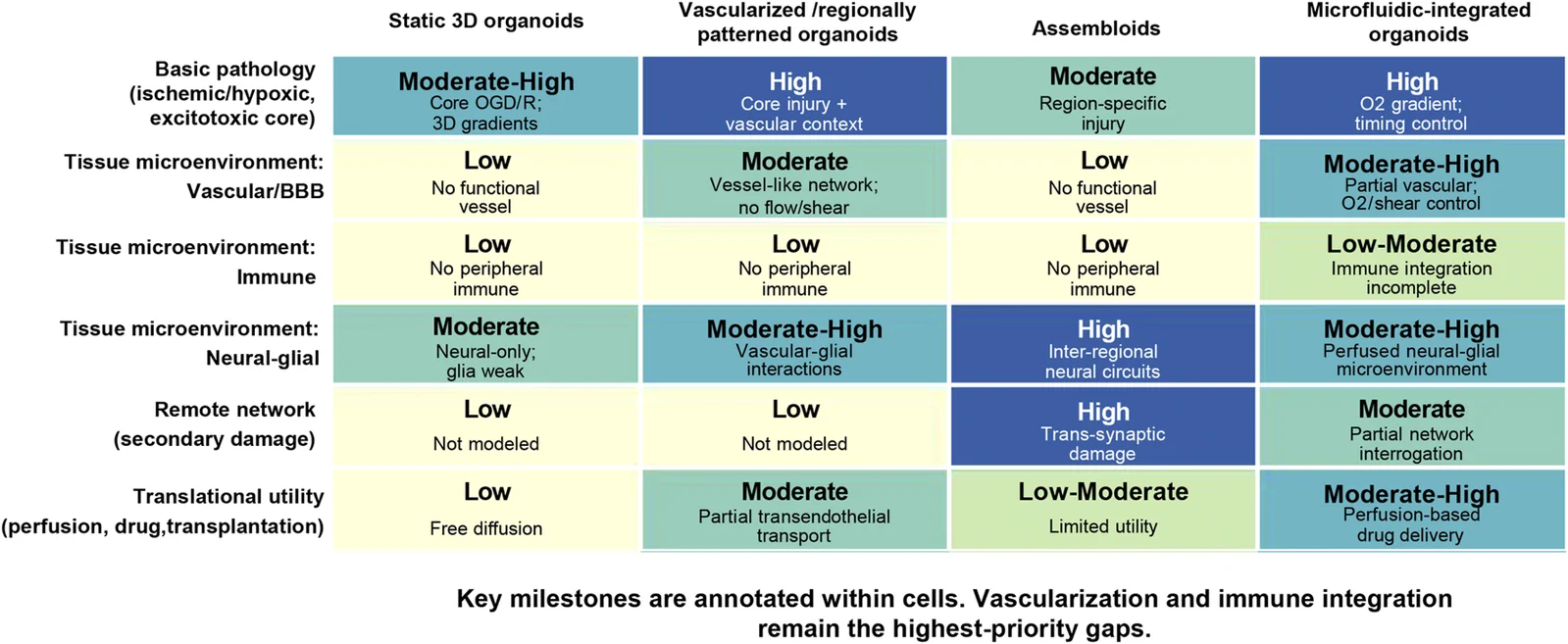
Demand–engineering strategy coupling matrix for brain organoids in stroke research. This framework summarizes how different organoid engineering strategies address key requirements in stroke modeling, including human-specific biology, tissue microenvironment, dynamic injury modeling, functional assessment, and translational evaluation. The matrix represents a qualitative assessment rather than a formal quantitative meta-analysis. Ratings were based on three predefined considerations: direct experimental evidence supporting the relevant capability, reproducibility across studies or experimental settings, and functional alignment between platform characteristics and stroke research requirements. “Low” indicates limited or indirect evidence; “Moderate” indicates partial capability with substantial limitations; and “High” indicates that a platform has demonstrated relatively strong capability under specific validated conditions, without implying complete recapitulation of physiological complexity. The organoid category encompasses a spectrum of models, including static cerebral organoids, multicellular organoids, and vascularized organoids. Higher ratings reflect capabilities demonstrated by more advanced organoid configurations rather than by early static models alone. Assembloids are mainly optimized for modeling inter-regional connectivity and cell-type interactions; therefore, their contribution to tissue microenvironment reconstruction differs from that of vascularized or microfluidic platforms. Microfluidic-integrated systems provide improved control over fluid flow, chemical gradients, and dynamic environmental parameters, enabling enhanced modeling of selected aspects of tissue-level interactions. This framework is intended to illustrate complementary strengths of different platforms rather than establish a strict replacement hierarchy. Future advances will likely involve integration of multiple engineering approaches to address distinct biological questions in stroke research.

**FIGURE 2:**
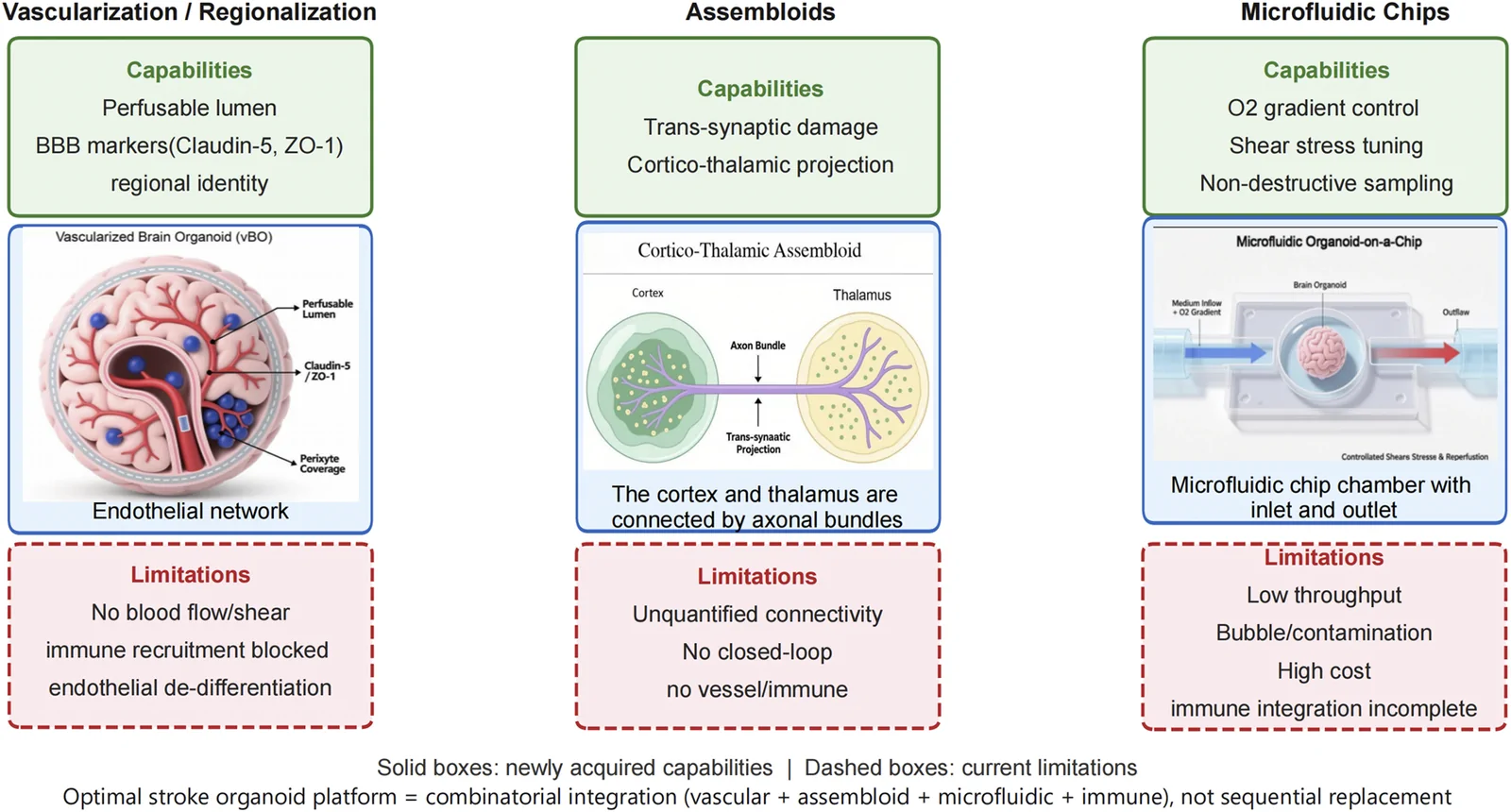
Capability–boundary framework of three technology iterations. Three generations are displayed in parallel columns: vascularization/regionalization, assembloids, and microfluidic chips. Upper solid boxes: newly acquired capabilities. Middle schematics: core structural/functional features. Lower dashed boxes: unresolved limitations. This framework emphasizes that each advance carries specific trade-offs, and progress lies in strategically combining strengths while addressing constraints. Each iteration solves a subset of prior limitations but introduces new constraints, suggesting that the optimal stroke organoid platform will likely require combinatorial integration rather than sequential replacement.

**FIGURE 3:**
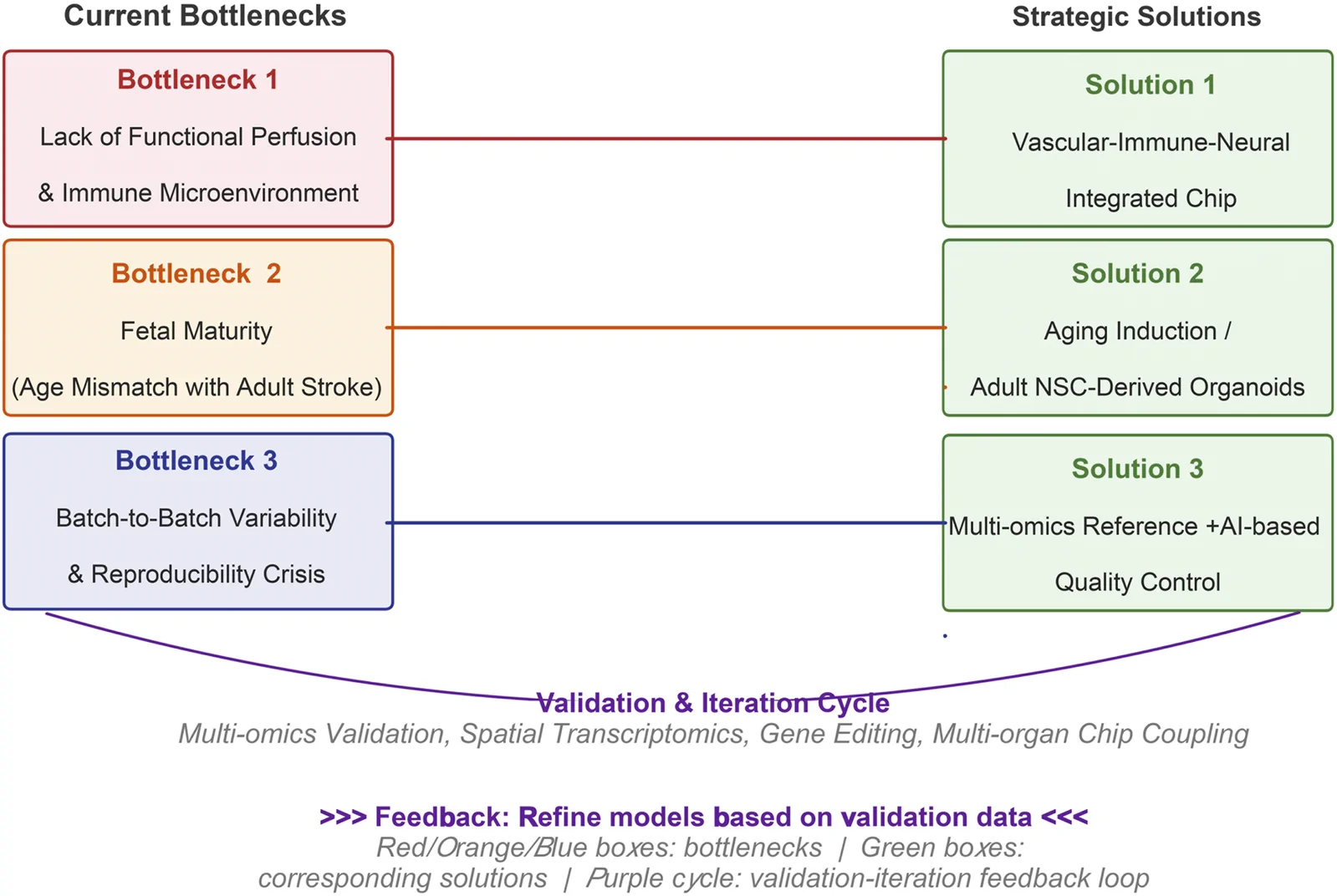
Core bottlenecks and strategic solutions. Left column: three fundamental bottlenecks—(i) lack of functional perfusion/complete immune microenvironment, (ii) fetal-stage maturity causing age mismatch with adult stroke, (iii) batch-to-batch variability/reproducibility crisis. Right column: corresponding solutions—(i) vascular–immune–neural integrated chip with microfluidics and immune cell circulation, (ii) aging induction or adult NSC derivation, (iii) multi-omics reference atlas with AI-based QC. Horizontal arrows map each bottleneck to its solution. Bottom curved arrow: validation–iteration feedback loop through multi-omics, spatial transcriptomics, gene editing, and multi-organ chip coupling.

### 1.4 Search strategy and scope

This is a narrative, non-systematic review. We searched PubMed, Web of Science, and Scopus through August 2026 using combinations of the following terms: brain organoids, cerebral organoids, assembloids, organoid-on-a-chip, microfluidics, stroke, ischemic stroke, hemorrhagic stroke, oxygen–glucose deprivation, OGD/R, neuroinflammation, PANoptosis, ferroptosis, SRGAP2C, electrophysiology, multi-electrode array, and transplantation. We prioritized primary research articles, authoritative reviews, and recent methodological papers. No formal inclusion/exclusion screening, risk-of-bias assessment, or meta-analysis was performed. Therefore, the review may be subject to selection bias and publication bias. The manuscript is organized into three main parts: (i) organoid engineering for stroke modeling; (ii) human-specific mechanisms revealed by organoids; and (iii) translational positioning, bottlenecks, and future directions.

## 2 *In vitro* reconstruction of stroke pathology models

### 2.1 Ischemic stroke modeling: the OGD/R parameter space

Oxygen–glucose deprivation/reoxygenation (OGD/R) remains the most widely applied ischemia-mimicking protocol: organoids are transferred from standard culture into glucose-free balanced salt solution under hypoxia (De Paola et al., 2023; Wang et al., 2023). The intrinsic oxygen gradients and nutrient diffusion limitations within 3D structures confer a spatiotemporal injury logic distinct from monolayer cultures.

Parameter variability spans orders of magnitude across laboratories: O_2_ concentrations 0.1%–5%, ischemic duration 2–24 h, reoxygenation 6–72 h (Gaston-Breton et al., 2023). At 1% O_2_ for several hours, PI-positive cells increase markedly, with further deterioration upon reoxygenation, confirming prominent reperfusion injury in 3D systems (De Paola et al., 2023). At 5% O_2_, cell viability loss is substantially attenuated—because actual oxygen partial pressure within 3D cores drops below 0.5%, generating “core necrosis, peripheral survival” spatial heterogeneity absent in 2D (Gaston-Breton et al., 2023; Vijaykumaran et al., 2026). This spatial pattern resembles the clinical concept of ischemic core and penumbra defined by DWI/PWI mismatch in acute stroke patients, although organoids provide only a reductionist surrogate (Kidwell et al., 2003). Extreme hypoxia may trigger stronger HIF-1α signaling, though correspondence with specific death modes remains unresolved (Li et al., 2025).

Glucose deprivation methodology is equally inconsistent: glucose-free DMEM substitution for EBSS may leave residual glucose (∼0.5 mM) sufficient to sustain glycolysis, compromising baseline ATP depletion (Kulkarni et al., 2020). Genetic background further confounds—organoids from different iPSC lines exhibit up to threefold differences in OGD/R sensitivity, serving both individualized testing and undermining reproducibility (Van Breedam et al., 2022).

Mechanistically, OGD/R engages multiple death pathways with temporal specificity. Caspase-3-activated apoptosis dominates early reoxygenation (≤6 h). Ferroptosis markers, including GPX4 downregulation and lipid peroxidation, emerge mainly between 6 and 24 h and may persist beyond 24 h; direct timing evidence in stroke organoids remains limited. Necroptosis (p-RIPK3/p-MLKL) during ischemia lacks robust direct evidence in organoid OGD/R (Mei et al., 2025; Kim et al., 2021). Death signals may propagate as waves from core outward. The timeline in Figure 4 should be regarded as a proposed model rather than a directly established organoid finding.

**FIGURE 4:**
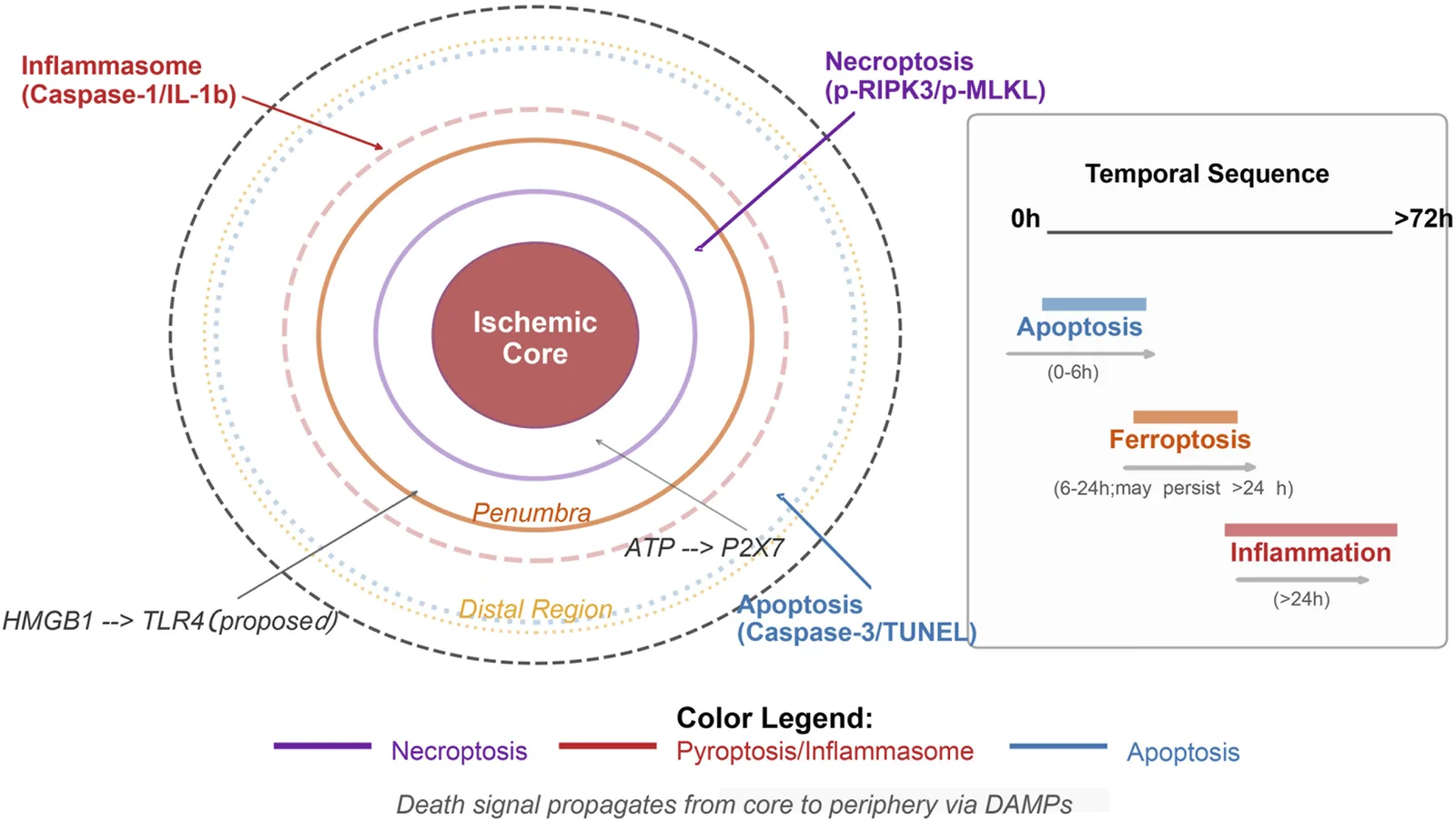
Proposed/hypothetical spatiotemporal dynamics of PANoptosis-like death signaling after OGD/R. Left: schematic organoid cross-section illustrating wave-like death signal propagation from ischemic core (dark red) through penumbra (orange) to distal region (pale yellow). Three concentric waves are proposed: necroptosis (purple, inner), inflammasome/pyroptosis (red, middle), and apoptosis (blue, outer). Inter-wave arrows indicate proposed DAMP-mediated propagation via ATP–P2X7 and HMGB1–TLR4. Right: proposed temporal sequence—apoptosis predominates ≤6 h; ferroptosis markers emerge 6–24 h and may persist beyond 24 h (GPX4↓, lipid peroxidation); sustained inflammation >24 h. This figure is a proposed/hypothetical model, not a directly established finding in stroke organoids. Direct evidence for PANoptosis in organoid stroke models is currently lacking.

Excitotoxic modeling employs exogenous glutamate (10–100 μM) or NMDA (50–200 μM, 0.5–4 h) (Bauersachs et al., 2022). A critical caveat: functional NMDA receptor expression in organoids reaches only 10%–20% of adult brain levels, as organoids remain fetal-stage, making excitotoxic responses inherently weaker than *in vivo* (Logan et al., 2020). This gap is instructive—the failure of NMDA antagonists may stem partly from human fetal-stage neurons’ stronger endogenous calcium-buffering capacity, which organoids inadvertently foreground, imposing an important demand: developmental stage must be matched (Ikonomidou and Turski, 2002). Additionally, under ischemia, astrocytic EAAT2 (GLT-1) reverses operation—ceasing uptake and releasing glutamate, amplifying excitotoxicity (Rossi et al., 2000). The full parameter spectrum is summarized in Figure 5 (left panel).

**FIGURE 5:**
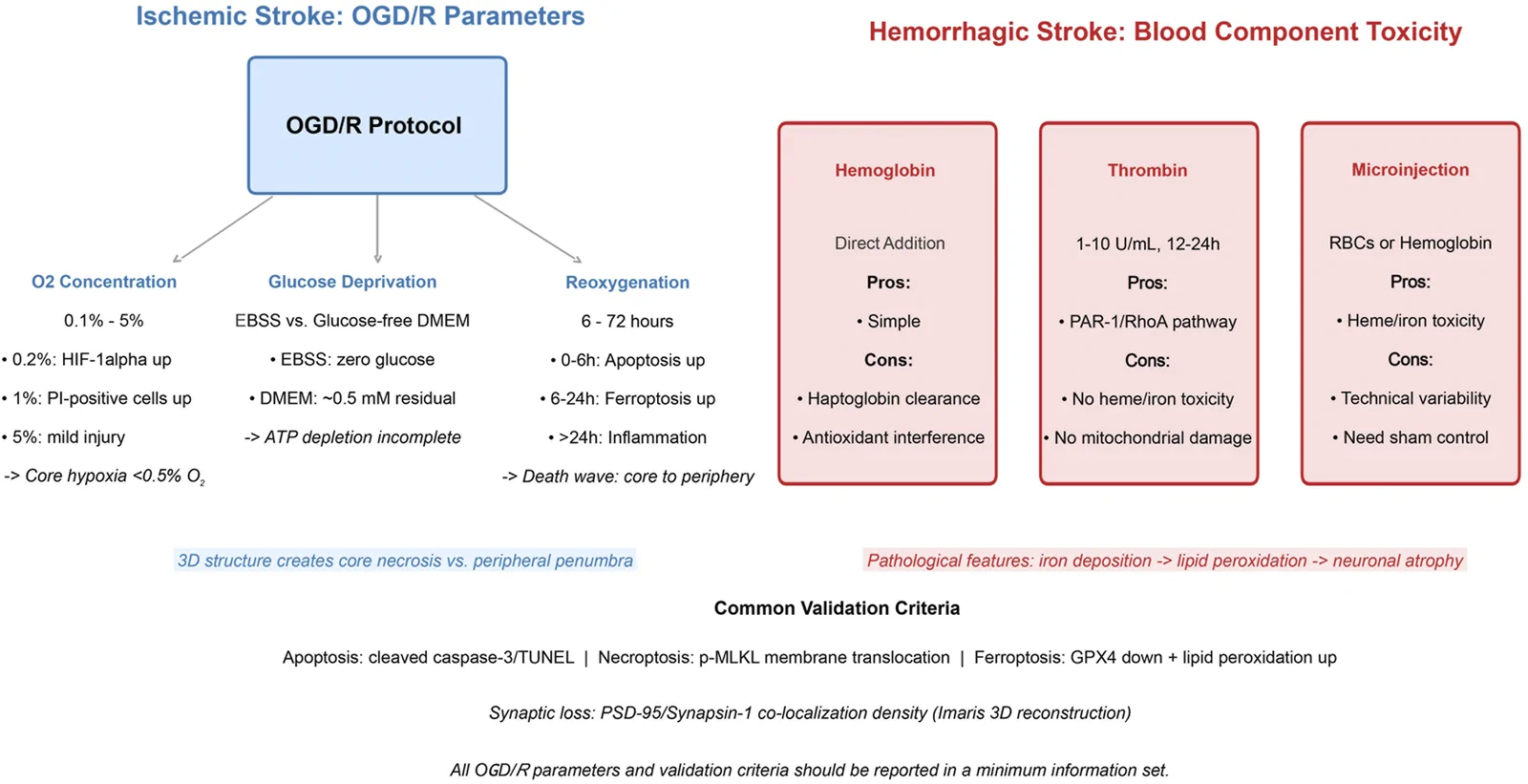
Modeling ischemic and hemorrhagic stroke pathology in brain organoids. Left: key OGD/R parameter variables—O_2_ concentration (0.1%–5%), glucose deprivation media (EBSS vs. glucose-free DMEM), reoxygenation duration (6–72 h). 3D architecture creates intrinsic oxygen gradients (core <0.5% O_2_), yielding spatially heterogeneous injury. Right: three hemorrhagic modeling approaches—direct hemoglobin addition, thrombin treatment (1–10 U/mL, 12–24 h, PAR-1/RhoA signaling), and microinjection—with respective advantages/limitations. Bottom: common validation criteria (cleaved caspase-3/TUNEL, p-MLKL, GPX4/lipid peroxidation, synaptic density via Imaris 3D reconstruction). The 3D oxygen gradient effect—producing core–penumbra heterogeneity—is an important distinction from 2D cultures and should be considered a quality-control consideration for stroke organoid models.

### 2.2 Hemorrhagic stroke modeling

Hemorrhagic modeling faces two challenges: blood component complexity and absent functional vasculature. Three approaches exist. Hemoglobin addition simulates free hemoglobin neurotoxicity following subarachnoid hemorrhage (Seah et al., 2025); a significant flaw is that organoid culture media contain substantial antioxidants, which systematically underestimate iron-driven oxidative damage. Thrombin treatment is pathophysiologically more relevant—administering 1–10 U/mL thrombin for 12–24 h produces dendritic retraction and astrocytic GFAP upregulation via PAR-1/RhoA/ROCK signaling, causing measurable tissue contraction (Caliaperumal et al., 2014; Ruiz-Loredo et al., 2011). This model misses heme/iron-mediated mitochondrial attack. Microinjection of red blood cell suspensions better captures chemical toxicity, enabling sequential observation of necrotic core formation and iron deposition, though sham-injection controls are indispensable for baseline calibration. Comparative strategies are summarized in Figure 5 (right panel).

### 2.3 Model validation

Validation requires multi-layered criteria: apoptosis (cleaved caspase-3/7, TUNEL), necroptosis (p-MLKL membrane translocation), and ferroptosis (GPX4 loss + lipid peroxidation probe) (Liu et al., 2026; Fei and Leng, 2026). A qualified OGD/R model should simultaneously demonstrate significantly increased PI + cells, elevated extracellular ATP, and reduced presynaptic synapsin I immunoreactivity (De Paola et al., 2023). Synaptic loss quantification employs Imaris 3D reconstruction for unbiased PSD-95/Synapsin-1 double-positive puncta counting (Gcwensa et al., 2026). Glial responses in organoids are substantially weaker than *in vivo* due to absent peripheral immune infiltration—conferring a “clean” advantage for isolating neuron-autonomous responses but systematically underestimating inflammatory amplification.

## 3 From static to dynamic: three-tier iterative upgrading

### 3.1 Vascularized brain organoids (vBOs)

Introducing HUVECs or iPSC-derived endothelial cells during differentiation generates CD31^+^ vascular-like networks with continuous tight junctions, partial pericyte coverage, and expression of Blood–brain barrier (BBB) signature proteins Claudin-5, ZO-1, Glut-1. Co-culture for weeks yields vascular plexuses; upon transplantation into immunodeficient mouse cortex, host vessels invade and anastomose, achieving functional perfusion (Shi et al., 2020; Cakir et al., 2019).

Vascularization contributes at three levels. First, it enables *in vitro* “microcirculatory failure” modeling—post-OGD/R, endothelial cells upregulate E-selectin and ICAM-1, exhibiting a no-reflow phenomenon (Eltaibany et al., 2026). Second, vBOs achieve Transendothelial electrical resistance (TEER) values of 150–300 Ω cm^2^ (below *in vivo* but superior to 2D co-culture), with post-OGD/R TEER drops mediated by MMP-9-dependent tight junction degradation (Fan et al., 2025). Third, drug delivery evaluation advances from free diffusion to partial transendothelial transport (Song et al., 2026).

Limitations are substantial: overall vascular permeability remains elevated; endothelial cells de-differentiate during prolonged culture. Critically, vBOs lack blood flow shear stress (∼10–20 dyn/cm^2^ *in vivo*)—the essential mechanical signal for BBB phenotype maintenance (Konig et al., 2025). During reperfusion, shear stress surges trigger adhesion molecule upregulation and tight junction rearrangements. In vBOs, the temporal chain—“shear drop → endothelial activation → immune recruitment”—is truncated; observed inflammatory responses reflect mainly chemical stress, not integrated hemodynamics. This may explain why anti-ICAM-1 strategies screened in vBOs have shown limited predictive value.

### 3.2 Regionally specified organoids and assembloids

Stroke is never a local event: cortical infarction triggers secondary thalamic atrophy, callosal Wallerian degeneration, and even functional reorganization of contralateral motor cortex—a phenomenon documented in clinical imaging studies of post-stroke remote neurodegeneration (Ong, 2022; Brodtmann et al., 2020). Regionally specified organoids—generated through spatiotemporal signaling modulation—express region-specific transcription factors and exhibit corresponding neurotransmitter phenotypes (Xiang et al., 2020; Miura et al., 2020).

Assembloids juxtapose organoids of different regional identities, inducing functional long-range connections. Cortico-thalamic assembloids show observable axonal bundles; cortical electrical stimulation evokes thalamic action potentials displaying synaptic transmission characteristics (Patton et al., 2024). This platform uniquely enables direct *in vitro* observation of anterograde and retrograde trans-synaptic degeneration: localized cortical OGD damages cortical neurons while thalamic-side neurons undergo secondary death—partially reversible by axonal conduction blockers—a phenomenon not documented in 2D co-culture (Lombardozzi et al., 2025). Such findings provide a potential mechanistic correlate to the progressive thalamic atrophy observed in clinical stroke populations (Brodtmann et al., 2020).

Key limitations: connection specificity—cortical axons may erroneously project to non-matching targets, and the extent to which heterologous connectivity compromises “remote damage” conclusions remains unquantified. Assembloids lack sensory input and feedback loops, precluding closed-loop thalamo-cortico-thalamic oscillations, directly limiting interpretive depth for network-level syndromes. They are better positioned as discovery tools than validation platforms.

### 3.3 Organoid-on-a-chip and microfluidic integration

Microfluidic technologies address the absence of kinetic simulation through three advances. First, oxygen gradient control: chips establish linear O_2_ gradients (1%–21%), enabling more precise injury threshold determination (Santiago et al., 2023). Second, precise ischemia–reperfusion timing: microvalve switching achieves complete perfusate exchange within tens of seconds. A notable finding: cortical organoids subjected to ≤2 h OGD followed by reperfusion recovered well; OGD exceeding 6 h before reperfusion caused sharp viability decline. This threshold is an experimental analogy to, but not a direct readout of, the clinical thrombolysis time window, which varies by modality and patient selection (Wu et al., 2025; Saver, 2006). Third, shear stress introduction: micro-pump-driven perfusion provides endothelial cells with mechanical signals important for BBB phenotype maintenance, partially compensating vBO structural deficits (Chen et al., 2025).

Engineering design principles are also advancing. Microfluidic devices can be classified by flow regime, channel geometry, and sensing integration. Oxygen gradient generators typically use gas-permeable materials and parallel channels to impose stable gradients; perfusion chips require careful control of shear stress, bubble formation, and dead volume. Recent work on brain organoids-on-chip has emphasized programmable interfaces, hydrogel encapsulation, and real-time readouts (Zhang et al., 2025a; Zhang et al., 2023; Zhang et al., 2025b; Zhang et al., 2025c; Zhang et al., 2026; Liu et al., 2021; Zhang et al., 2022). For example, platinum wire-embedded devices allow interior signal recording from neural spheroids (Zhang et al., 2025a), while programmable peptide interfaces and core-shell hydrogel microcapsules improve on-demand neural culturing and cell encapsulation (Zhang et al., 2026; Liu et al., 2021). Lab-on-chip systems for *ex vivo* neuronal networks provide additional design precedents for multi-electrode array integration and fluidic control (Zhang et al., 2022). These engineering advances are important because stroke is a dynamic process involving flow cessation, reperfusion, shear changes, and immune cell recruitment; static culture cannot reproduce this kinetic complexity.

Beyond engineering, organoid-on-a-chip introduces “temporal biology”—non-destructive sequential sampling from the same organoid constructs can generate a more complete pathological time series, reducing inter-batch variation. High barriers persist: equipment costs, limited throughput, and risks of bubbles/contamination during prolonged operation position these systems as exploratory frontier tools rather than routine screening platforms (Figure 2).

### 3.4 Electrophysiological recording and functional recovery in organoid stroke models

Stroke pathophysiology critically involves network-level electrical dysfunction. However, electrophysiological readouts in brain organoid stroke models remain underdeveloped. Most OGD/R studies rely on viability, morphology, and biochemical endpoints, with limited use of multi-electrode arrays (MEAs), patch-clamp, or implantable microelectrodes. This is a major gap because neuronal death and synaptic loss do not necessarily track network failure. Recent methodological advances may help. Platinum wire-embedded culturing devices allow interior signal recording from lollipop-shaped neural spheroids (Zhang et al., 2025a). Single-neuron MEA chips enable manipulation and analysis of individual neurons within networks (Zhang et al., 2023). Magnetic nanoparticle-based single-neuron manipulation has been used to design customized neural circuits (Zhang et al., 2025b). Brain organoids-on-chip further offer opportunities for combining perfusion, oxygen control, and electrophysiological readout (Zhang et al., 2025c). Incorporating these approaches into stroke organoid models could define “electrophysiological penumbra,” detect seizure-like discharges after OGD/R, and assess functional recovery after transplantation. At present, such data are sparse, and electrophysiological recovery should be framed as a priority for future validation rather than an established organoid capability.

## 4 Human-specific dimensions revealed by brain organoids

The phenomena described below are working hypotheses based on organoid observations unless otherwise stated. “Human-specific” denotes phenomena observed in human organoids that are inadequately documented in animal models. We incorporate corresponding animal model evidence as reference (Table 1).

**TABLE 1:** Human-specific mechanisms revealed by brain organoids in stroke research.

Dimension	Organoid findings	Differences from animal/2D models	Translational implications
A. Findings supported by organoid observations, with animal/2D comparison			
Microglial response	OGD/R induces DAM-like state; sustained TNF-α/il-1β; no clear restorative transition	Rodent models show more classical M1–M2 switch; human microglia show persistent activation	Human neuroinflammatory trajectories may inform anti-inflammatory strategies; DAM signatures are candidate biomarkers
Astrocytic response	SASP-like phenotype: p21/p16+, IL-6/il-8/mcp-1 release	Rodent astrocytes often show A1/A2 polarization; human astrocytes show senescence-dominant reactivity	SASP/senolytics may be more relevant than A1/A2 modulation in human stroke
Complement-mediated pruning	C1q/C3 deposition; CR3-mediated engulfment; prolonged synaptic loss	Rodent pruning is more transient; human organoids show prolonged/exaggerated loss	C3a receptor antagonists are candidate therapies; prolonged pruning may underlie persistent cognitive deficits
Synaptic regulation by human-specific genes	SRGAP2C downregulation after OGD/R is associated with spine/mEPSC deficits; NOTCH2NL may inhibit NeuroD1	SRGAP2C, ARHGAP11B, NOTCH2NL are absent in rodents	Human-specific genes are candidate neurorepair targets; rodent rehabilitation strategies may need re-evaluation

Hypothesis	Current evidence	Required validation
B. Hypotheses awaiting validation		
PANoptosis-like propagation waves	No direct stroke-organoid evidence; Figure 4 is hypothetical; extrapolated from non-organoid systems	ZBP1/RIPK3 knockout organoids; live imaging; single-cell death-pathway mapping
ARHGAP11B in post-stroke neurogenesis	Sparse clues; mainly developmental cortex data	Adult/aged organoid models; lineage tracing; loss-of-function
NOTCH2NL–NeuroD1 axis in neuroblast migration	Upregulation after OGD/R reported; causal role unclear	CRISPR perturbation; migration assays; *in vivo* confirmation

**Abbreviations:** DAM, disease-associated microglia; DAMP, damage-associated molecular pattern; GPX4, glutathione peroxidase 4; HMGB1, high-mobility group box 1; IL, interleukin; mEPSC, miniature excitatory postsynaptic current; MCP-1, monocyte chemoattractant protein-1; OGD/R, oxygen–glucose deprivation/reoxygenation; SASP, senescence-associated secretory phenotype; TLR4, Toll-like receptor 4; TNF-α, tumor necrosis factor alpha.

### 4.1 Neuroinflammation: disease-associated microglial (DAM) states and astrocytic senescence

Microglia are introduced via exogenous iPSC-derived precursors or endogenous directed differentiation (Schafer et al., 2023). Human organoid microglia respond to OGD/R with a DAM-like state: pro-inflammatory genes (TNF-α, IL-1β) are upregulated early in reoxygenation but, rather than transitioning to a reparative phenotype, sustain a persistent pro-inflammatory transcriptomic profile (Yi et al., 2025). This sustained activation pattern contrasts with the transient M1/M2 switch documented in rodent stroke models and may partially explain the protracted neuroinflammatory trajectory observed in some human patients.

Complement-mediated synaptic pruning constitutes a second dimension. In human organoids, C1q/C3 deposit on presynaptic terminals post-reoxygenation, driving microglial CR3-mediated excessive engulfment; synaptic density at 48 h post-reoxygenation falls >50% compared to controls, with C3a receptor antagonists significantly rescuing this loss (Sun et al., 2022; Liu et al., 2024a). This pruning persists longer and at greater intensity than in rodents—where complement deposition resolves within about 1 week—offering a mechanistic candidate for the clinical impression that post-stroke cognitive impairment is more persistent in humans (Liu et al., 2024a).

Astrocytic responses do not segregate into neurotoxic A1/neuroprotective A2 subtypes but exhibit a senescence-associated secretory phenotype (SASP)—p21/p16INK4a upregulation with sustained IL-6/IL-8/MCP-1 release (Aguado et al., 2021). If confirmed *in vivo*, continuing to use the A1/A2 dichotomy for anti-inflammatory drug screening may systematically miss SASP as a key human therapeutic target.

### 4.2 PANoptosis propagation waves: a proposed model awaiting validation

The PANoptosis concept proposes that apoptosis, necroptosis, and pyroptosis can be simultaneously activated, assembling into PANoptosome complexes (ZBP1, RIPK3, CASP8, ASC) (Chen et al., 2023). Direct evidence in stroke organoids remains absent. The following represents extrapolation from other systems and should be regarded as a hypothesis-generating model, not an established finding. Figure 4 is therefore labeled as a proposed/hypothetical model.

3D transparency and live-cell imaging provide a potential window for testing this hypothesis. Sequential activation of inflammasome, necroptotic execution, and apoptotic signals has been observed during reoxygenation in some systems, with overlapping features detectable in specific regions and death signals expanding in wave-like patterns from the injury center outward (Figure 4). This wave-like propagation has not been recorded in real time in animal models. In non-organoid systems, PANoptic cells release extracellular ATP (acting on P2X7) and HMGB1 (acting on TLR4) to induce neighboring cells into the same death program (Tang et al., 2026). If reduced death propagation were observed in ZBP1-KO or RIPK3-KO organoids, it would provide stronger evidence for the PANoptosis causal chain. A technical boundary: current models cannot completely distinguish “genuine cell-to-cell propagation” from bystander effects.

### 4.3 Human-specific genes in synaptic plasticity impairment: the SRGAP2C paradigm

SRGAP2C, a human-specific gene duplication product, encodes a truncated antagonist of full-length SRGAP2 that promotes dendritic spine maturation and localizes to excitatory postsynaptic densities (Charrier et al., 2012). SRGAP2C is absent from the mouse genome—a prime example where animal models cannot directly address human post-stroke synaptic pathology.

The original functional studies established SRGAP2C in spine maturation (Charrier et al., 2012). A subsequent review of human-specific paralogs summarizes preliminary organoid observations that SRGAP2C expression increases with developmental progression and that, after OGD/R, its mRNA drops significantly, accompanied by decreases in spine density and mature mushroom-type spines (Libé-Philippot and Vanderhaeghen, 2021). These observations should be interpreted cautiously because Libé-Philippot and Vanderhaeghen (2021) is a review rather than a primary experimental source for all OGD/R phenotypes. CRISPRi knockdown of SRGAP2C, even without OGD stimulation, has been reported to produce spine density reduction and synchronous mEPSC frequency decrease, suggesting that post-stroke SRGAP2C downregulation may impair synaptic function independent of cell death. This finding provides a testable framework for why rodent-based rehabilitation strategies have shown limited human efficacy, but primary stroke-organoid validation is still needed.

Two additional human-specific genes have more nascent evidence. ARHGAP11B (absent in mice) promotes basal progenitor amplification in embryonic cortex; its role in post-stroke adult neurogenesis rests on sparse clues (Florio et al., 2015). NOTCH2NL, upregulated after OGD/R, inhibits NeuroD1 via Notch signaling, potentially hindering neuroblast migration to injury sites; causal validation in the stroke setting is still lacking. Collectively, human-specific mechanisms and animal model comparisons are summarized in Table 1.

## 5 Organoid-driven therapeutic strategy development

### 5.1 High-throughput screening and personalized testing—and the positioning of organoids in the preclinical pipeline

Organoid-based screening may serve as an “intermediate filter layer” between 2D screening and animal experiments. Ferrostatin-1 robustly improves post-OGD/R viability in 2D yet shows markedly attenuated effects in cortical organoids, with off-target effects emerging at elevated doses (Tschuck et al., 2024). Patient-derived iPSC organoids enable “patient avatar” drug response prediction; however, 3–6-month generation/maturation timelines remain a substantial barrier for acute stroke treatment.

Beyond individual applications, it is important to position organoids within the broader preclinical pipeline for stroke drug development. We propose that organoids currently occupy a distinct, bounded niche.

#### 5.1.1 Scenarios where organoids can partially replace animal experiments

(i) Early-stage validation of human-specific molecular targets (e.g., SRGAP2C or NOTCH2NL modulation) that are absent from rodent genomes and cannot be tested in conventional species. (ii) CRISPR-based functional screening of candidate risk genes identified by human genome-wide association studies for stroke susceptibility. (iii) Individualized drug sensitivity testing on patient-derived organoids to predict idiosyncratic neurotoxicity or neuroprotection, thereby avoiding futile animal experiments in non-responder genotypes. In these contexts, organoids provide human-specific information that animals cannot offer.

#### 5.1.2 Scenarios where organoids cannot replace animal experiments

(i) Assessment of blood–brain barrier permeability with complete systemic pharmacokinetics—current vBOs lack the enzymatic and transport maturity of adult BBB and have no connection to liver or kidney clearance. (ii) Full-scale evaluation of immune-mediated secondary injury—post-stroke infiltration of neutrophils, monocytes/macrophages, and T lymphocytes constitutes a dominant driver of subacute-to-chronic tissue damage, and organoids lack a complete circulating immune system. (iii) Long-term functional behavioral recovery assays—recovery of sensorimotor function or cognitive domains requires intact neural circuitry with sensory input and behavioral output, which organoids by definition cannot provide. For these complex system-level endpoints, well-designed animal models remain irreplaceable.

#### 5.1.3 A proposed three-stage screening funnel

Stage 1—2D high-throughput screening (entry: any chemical library; exit: confirmed efficacy in >3 iPSC lines without cytotoxicity). Stage 2—organoid-based validation (entry: hits from Stage 1; exit: demonstrated target engagement in 3D tissue, absence of unexpected neurotoxicity, and, if applicable, human-specific pathway modulation). Stage 3—animal model confirmation (entry: candidates that show organoid efficacy and lack obvious red flags; exit: *in vivo* pharmacokinetics/pharmacodynamics, systemic safety, immune interaction, and behavioral recovery assessed in at least two species with comorbid conditions). Each stage has transparent entry and exit criteria, and agents failing at Stage 2 are deprioritized before animal testing.

### 5.2 Biomarker discovery

Materials released into organoid culture supernatant can be compared to patient cerebrospinal fluid or serum. Neuron-derived exosomes surge after OGD/R, with decreased miR-124 and increased miR-21 (Liu et al., 2024b); proteomic analysis identifies candidate proteins (UCHL1, SNAP25 fragments) upregulated exclusively post-OGD/R. Neurofilament light chain exhibits a biphasic release pattern—slow ischemia-phase release, sharp early-reoxygenation elevation, secondary late-reoxygenation plateau—not clearly captured *in vivo* because of blood clearance interference (De Paola et al., 2023). Relative fold-changes and release rates are more informative than direct concentration comparisons. These findings remain exploratory and require cross-validation in patient samples.

### 5.3 Organoid transplantation

Transplanting organoids as “living repair materials” into stroke animal models has produced positive evidence: grafts integrate within weeks; transplanted neurons extend long-range projections to contralateral cortex, thalamus, and spinal cord; partial motor recovery is documented; spontaneous EPSCs and host-stimulation responses suggest functional synaptic integration (Ondriš et al., 2025; Cao et al., 2023; Kitahara et al., 2020).

Controversies are equally significant: grafts may exhibit “overgrowth” from persistent neural progenitor proliferation, compressing host tissue (Kitahara et al., 2020); paroxysmal spike discharges and spontaneous seizures have been recorded, potentially from excitation–inhibition imbalance due to insufficient GABAergic interneuron proportions (Cao et al., 2023). Optimization strategies include co-transplanting cortical with medial ganglionic eminence organoids. Ethical constraints explicitly prohibit non-human primate transplantation; graft volume must not exceed 5% of host brain volume. Organoid transplantation studies will likely remain limited to rodent models in the near term.

## 6 Current bottlenecks and future directions

### 6.1 Triple technological bottlenecks—stated with candor

The translational utility of organoids is currently capped by three fundamental bottlenecks.

#### 6.1.1 Bottleneck 1: absent functional perfusion and complete immune microenvironment

Current vBO endothelial plexuses lack blood flow and shear stress; BBB properties reach only a fraction of *in vivo* levels (Tan et al., 2023). More critically, inflammatory responses in organoids reflect almost exclusively central innate immunity. In actual stroke, a large fraction of secondary brain injury is driven by peripheral immune cells—neutrophils arriving within hours, followed by monocytes and T lymphocytes over days—a cascade that organoids cannot fully reproduce (Gelderblom et al., 2009). Consequently, anti-inflammatory strategies screened in current organoid systems address mainly the initial, neuron-autonomous phase of injury and may miss immune-mediated pathology of subacute and chronic stroke. Organoids are therefore suitable for studying early neuronal autonomous death, but inappropriate for modeling the secondary immune amplification that constitutes much of clinical post-stroke deterioration.

#### 6.1.2 Bottleneck 2: “fetal-stage” maturity—modeling infant brain responses in an adult disease

A direct comparison reveals the discrepancy: cortical organoid gene expression profiles correspond to human fetal weeks 12–20 (Logan et al., 2020), whereas the average age of stroke onset is approximately 70 years (GBD 2021 Stroke Risk Factor Collaborators, 2024). In essence, organoid-based stroke models simulate how a fetal/early developmental brain may respond to ischemia, not how an aged brain does. The aged brain is characterized by mitochondrial decline, autophagic flux blockage, chronic low-grade inflammation, and accumulated DNA damage—features largely absent in fetal-stage organoids. As a result, injury responses may be underestimated, and neuroprotective agents identified in organoids may target pathways (e.g., developmentally upregulated NMDA receptor subtypes) that are substantially downregulated in the aged human brain. Screening and therapeutic endpoints thus point to two molecularly different landscapes.

#### 6.1.3 Bottleneck 3: standardization and reproducibility

Multi-center studies reveal that even with fixed iPSC lines and protocols, cortical organoids exhibit substantial neuronal-to-astrocytic ratio variability and high OGD/R injury metric coefficients of variation. Sources include Matrigel batch differences, incubator micro-oxygen fluctuations, and manual manipulation subjectivity (Glass et al., 2024). These three bottlenecks and their targeted solutions are mapped in Figure 3.

### 6.2 Future directions

#### 6.2.1 Immune–perfusion integration

The next-generation platform envisions a “vascular–immune–neural” tripartite organoid: introducing iPSC-derived microglia into vBOs while incorporating a peripheral blood mononuclear cell circulation module into the microfluidic chip, with programmable microvalves directing Peripheral blood mononuclear cells (PBMC) upon “stroke mode” initiation (Tan et al., 2023).

#### 6.2.2 Maturity catch-up

Aging induction via repetitive low-dose ionizing radiation or telomerase inhibitor treatment can induce p21 upregulation and DNA damage foci, pushing organoids toward “senescence-like” characteristics—quality-controlled by single-cell transcriptomics. More fundamentally, direct derivation from adult neural stem cells faces sample acquisition difficulties.

#### 6.2.3 Standardization infrastructure

A multi-omic reference atlas using scRNA-seq combined with spatial transcriptomics at key differentiation nodes can establish “molecular fingerprints,” calculating “maturity scores” and “regional purity scores” to admit only organoids within specified reference ranges. Machine learning algorithms predicting organoid quality from brightfield images could enable high-throughput non-destructive selection.

#### 6.2.4 Gene editing

For stroke-related single-gene risk loci, CRISPR-Cas9 editing efficiency in organoids has improved; base editing shows potential in repairing pathogenic mutations (e.g., NOTCH3), creating a “gene correction–organoid validation” closed loop for monogenic cerebral small-vessel diseases such as CADASIL.

#### 6.2.5 Multi-organ coupling

An emerging but technically challenging direction is the development of “body-on-a-chip” platforms, which connect brain, cardiac, and vascular organoid systems through microfluidic circulatory networks to investigate mechanisms such as microemboli formation in cardioembolic stroke. However, these approaches remain at an early conceptual and technical stage.

### 6.3 Limitations of this review

This review is narrative and non-systematic. Although we searched major databases and prioritized primary literature, we did not perform duplicate screening, formal risk-of-bias assessment, or quantitative synthesis. Selection bias, English-language bias, and publication bias are possible. Several cited 2025–2026 references are recent and may not yet have independent replication. Organoid stroke models are a fast-moving field; negative or null findings may be underrepresented. Finally, many “human-specific” mechanisms discussed here are based on organoid observations that still require *in vivo* validation. These limitations should be considered when interpreting the proposed framework.

## 7 Conclusion

The evolutionary trajectory of brain organoids in stroke research reflects a sustained dialogue between technological iteration and clinical demand (Figure 1). Organoids provide an experimentally accessible intermediate-complexity system in which human-specific mechanisms—including SRGAP2C-associated synaptic regulation, DAM-like state transitions, and proposed PANoptosis-related processes—can be investigated under controlled conditions. These approaches may complement existing models by providing insights into mechanisms that are difficult to capture in conventional experimental systems.

However, to achieve clinical impact, organoids must be deployed with clear-eyed awareness of their boundaries. Their current fetal transcriptomic signature and absent peripheral immunity mean that they faithfully recapitulate mainly the earliest, neuron-autonomous phase of ischemic injury—a critical but limited segment of total stroke pathology. Integrating perfusable vasculature, immune surveillance, electrophysiological circuitry, and standardized outcome measures remains important unfinished work. Simultaneously, establishing standardized evaluation frameworks—including unified differentiation protocols, quality-control criteria, injury parameter definitions, and efficacy evaluation “minimum information sets”—will be important for improving reproducibility and facilitating the translation of organoid platforms toward clinical applications. When organoid stroke data from multiple laboratories become directly comparable and meta-analyzable, brain organoids may embed themselves meaningfully in the drug development and personalized medicine decision chains for stroke.
